# Using literature-based discovery to identify candidate genes for the interaction between myocardial infarction and depression

**DOI:** 10.1186/s12881-019-0841-8

**Published:** 2019-06-11

**Authors:** Zhenguo Dai, Qian Li, Guang Yang, Yini Wang, Yang Liu, Zhilei Zheng, Yingfeng Tu, Shuang Yang, Bo Yu

**Affiliations:** 1 Department of Cardiology, The Second Affiliated Hospital of Harbin Medical University, Harbin, 150086 China; 2 The Key Laboratory of Myocardial Ischemia, Harbin Medical University, Ministry of Education, Harbin, China; 3 Department of Neurology, The Second Affiliated Hospital of Harbin Medical University, Harbin, 150086 China

**Keywords:** Myocardial infarction, Depression, BITOLA, Candidate genes, Text mining, Gene expression profiling

## Abstract

**Background:**

A multidirectional relationship has been demonstrated between myocardial infarction (MI) and depression. However, the causal genetic factors and molecular mechanisms underlying this interaction remain unclear. The main purpose of this study was to identify potential candidate genes for the interaction between the two diseases.

**Methods:**

Using a bioinformatics approach and existing gene expression data in the biomedical discovery support system (BITOLA), we defined the starting concept X as “Myocardial Infarction” and end concept Z as “Major Depressive Disorder” or “Depressive disorder”. All intermediate concepts relevant to the “Gene or Gene Product” for MI and depression were searched. Gene expression data and tissue-specific expression of potential candidate genes were evaluated using the Human eFP (electronic Fluorescent Pictograph) Browser, and intermediate concepts were filtered by manual inspection.

**Results:**

Our analysis identified 128 genes common to both the “MI” and “depression” text mining concepts. Twenty-three of the 128 genes were selected as intermediates for this study, 9 of which passed the manual filtering step. Among the 9 genes, *LCAT*, *CD4*, *SERPINA1*, *IL6*, and *PPBP* failed to pass the follow-up filter in the Human eFP Browser, due to their low levels in the heart tissue. Finally, four genes (*GNB3*, *CNR1*, *MTHFR*, and *NCAM1*) remained.

**Conclusions:**

*GNB3*, *CNR1*, *MTHFR*, and *NCAM1* are putative new candidate genes that may influence the interactions between MI and depression, and may represent potential targets for therapeutic intervention.

## Background

Myocardial infarction (MI) is a highly prevalent cardiovascular disease. The American Heart Association released a scientific statement in 2014 and recommended that depression should be considered a risk factor for adverse medical outcomes in patients with acute coronary syndrome [1]. Depression may cause many adverse outcomes, including autonomic dysfunction [2], inflammation [3], endothelial dysfunction [4, 5], hyperactivity of the hypothalamic-pituitary-adrenal axis [6], and poor compliance [7], which subsequently lead to an increased risk of MI. Both the severity and cumulative duration of depressive symptoms have a negative impact on the MI prognosis [8]. On the other hand, patients with MI may have a higher prevalence of depression [9]. In an assessment of 10,785 patients with MI performed using a structured clinical interview, depression was common and persistent in MI survivors. Major depression was identified in approximately 1 of 5 (19.8%) patients hospitalized with MI [10]. Thus, understanding the interaction between MI and depression is very important for the development of therapeutic interventions and determining patients’ needs.

The biomedical support discovery system (BITOLA) is a sophisticated bioinformatics tool that enables new discoveries, such as mining new information from the literature without using patient tissue samples, especially for identification of key candidates, and finding potentially new relationships among various biomedical concepts [11, 12]. Some researchers have used the text mining tools to identify candidate genes for diseases [13], such as multiple sclerosis and bilateral polymicrogyria [12, 14, 15]. In addition, using the BITOLA system, genes neural cell adhesion molecule 1 (*NCAM1*) and CD4 were identified as potential candidate genes in the interaction between depression and oral lichen planus [16].

Because the molecular mechanisms underlying the interaction between MI and depression remain unclear, the aim of the study is to identify new potential candidate genes linking these two diseases.

## Methods

### Extracting intermediate concepts from the BITOLA system

**Fig. 1:**
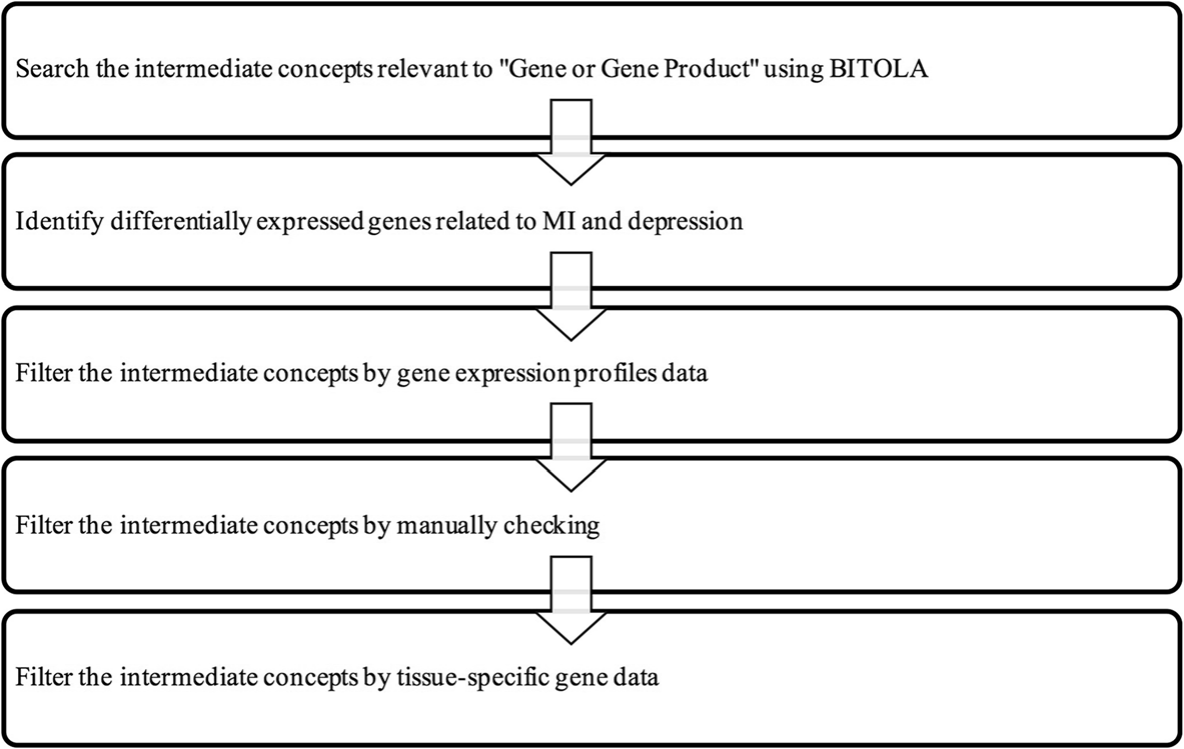
Flow chart of the study design

BITOLA is an interactive, literature-based, biomedical discovery support system (http://arnika.mf.uni-lj.si/pls/bitola2/bitola) [17]. The purpose of the system is to generate new findings by discovering potentially new relationships between biomedical concepts, especially candidate genes that have aetiological relationships with diseases. Currently, the set of concepts in the BITOLA includes Medical Subject Headings (MeSHs), which are utilized to index human genes from the Human Genome Organization (HUGO) and Medline [11]. By mining the Medline database, new information from the literature can be explored to identify new potential candidate genes linked to both MI and depression, and the potential new relationships can be discovered. Flow chart of the study design was shown in Fig. 1.

According to the proposed instructions of the tool, we used a closed discovery system in this study. Briefly, the item “Myocardial infarction” was entered as the starting concept X (Semantic types: disease or syndrome), and the items “Major Depressive Disorder” and “Depressive disorder” were entered as the end concepts Z (semantic types: Mental or Behavioral Dysfunction). Using those concepts, intermediate concepts Y were examined and extracted. In this study, the semantic types of intermediate concepts mainly referred to the “Gene or Gene Product”. Then, the intersection of the two gene sets of related concepts Y (gene or gene product) in total was retrieved for further analysis. These intermediate concepts were defined as the candidate intermediate molecules (CIMs).

### Identifying differentially expressed intermediate concepts

Next, we tentatively filtered and evaluated the “Gene or Gene Product” by overviewing their mRNA (messenger ribonucleic acid) expression levels under different conditions (MI vs. control or depression vs. control). We reserved differentially expressed “gene or gene product” for the next analysis and excluded non-differentially expressed genes.

### Gene expression datasets and statistical analysis

Gene expression datasets were obtained from the GEO database. The MI datasets used in this study are GSE48060, GSE83500, GSE97320, and GSE61145. GSE48060 was developed from the PBMCs of 52 patients diagnosed with MI and normal controls [18]. The GSE83500 dataset was developed from the aortic wall of MI patients and healthy individuals. GSE97320 and GSE61145 were developed from the peripheral blood from 6 and sera from 24 MI patients and normal controls. [19]. The depression datasets used in this study are GSE54562, GSE54563, GSE54564, GSE54565, GSE54566, GSE54567, GSE54568, GSE54570, GSE54571, GSE54572, and GSE54575 [20].

All GEO datasets were obtained from the GEO NCBI database, and the DEGs between the case group and the normal controls were analysed using the integrated GEO2R tool [21, 22]. Samples were assigned within a GEO series as either a normal control or case group depending upon the sample source and experimental classification. A T-test was used to sort out the DEGs. Multiple testing was applied using the Benjamini and Hochberg false discovery rate method. GEO2R provides a list of all probes (and corresponding gene aliases) ranked according to their degrees of differential expression. The top 250 probes were selected for the subsequent analysis, and finally the probes were converted into gene names.

### Manual checking of the intermediate concepts

False-positive genes may be identified during literature mining, and manually checking is a precise method to recognize these genes. We manually checked the gene symbols in the co-occurrence literature together with MI and depression and excluded the ambiguous terms that could apply to other topics.

### Evaluating expression patterns of the remaining “gene or gene product”

After manually checking the intermediate concepts, the remaining “Gene or Gene Product” were further filtered based on tissue-specific expression. For inclusion as candidate genes for the interaction of MI and depression, the genes from the list had to show a specific pattern of expression in both the heart and brain tissue; genes that did not satisfy the conditions were excluded. The Human eFP (“electronic Fluorescent Pictograph”) Browser (http://bar.utoronto.ca/efp_human/) was used to rapidly interpret the gene expression profiles; this program enables the user to easily visualize large-scale data sets based on representations of the human body [23]. In the gene expression profiling studies, the gene symbol was entered, the “Absolute” mode was chosen for interpretation, and the “Nervous” or the “Circulatory Respiratory” data source was selected. After clicking “Go”, the representations of human samples are coloured based on the expression level of the gene of interest to generate expression “anatograms” for rapid interrogation. Using this procedure, we can determine whether the given “Gene or Gene Product” is most strongly expressed in the heart or brain tissue. A yellow-red scale is used depict the expression levels, with yellow denoting no expression in a given depiction of a tissue and red denoting maximal expression [23].

## Results

### Intermediate concepts relevant to “Gene or Gene Product” for MI and depression

Using the adapted discovery algorithm with the starting concept X and end concept Z and its integration into the closed BITOLA system, we searched the entire intermediate concept Y relevant to “Gene or Gene Product”. We defined the starting concept X as “Myocardial Infarction” and end concept Z as “Major Depressive Disorder” or “Depressive disorder”. In this manner, 72 and 111 “gene or gene product” were suggested by the closed BITOLA system with the starting concept “Myocardial Infarction” and the end concepts “Major Depressive Disorder” and “Depressive disorder”, respectively. The intersection of the two gene sets of 128 related concepts Y (gene or gene product) in total was selected for further analysis, and we defined these selected genes as the CIMs.

### Genes differentially expressed in both MI and depression

Analysis of the GSE48060, GSE83500, GSE97320, and GSE61145 for MI, GSE54562, GSE54563, GSE54564, GSE54565, GSE54566, GSE54567, GSE54568, GSE54570, GSE54571, GSE54572, and GSE54575 data sets for major depressive disorders obtained from the Gene Expression Omnibus (GEO) revealed 2750 differentially expressed genes (DEGs). After contrastive analysis, seven genes (*IL-6, HLA-B, PPBP, PTPRC, SERPINA1, RERE,* and *PADI4*) were found to overlap between the 128 CIMs and the DEGs from GSE83500, GSE97320, and GSE61145. Meanwhile, sixteen genes (*FCGR3B, LPA, STAR, ESR1, GNB3, PAG1, NSF, ESD, LCAT, DMD, AR, CNR1, CPAMD8, HLA-B, MTHFR, and NCAM1*) overlapped between the 128 CIMs and the DEGs from GSE54563, GSE54564, GSE54565, GSE54567, GSE54568, GSE54571, and GSE54572 (Table 1). We further explored the correlations between MI and depression by defining the overlap between the DEGs and the 128 CIMs (Tables 1 and 2).

**Table 1:** Description of the 11 MI and MDD microarray platforms and the gene symbols that overlapped with the CIMs

Disease	Series	Tissue	Platform	Control samples (n)	Subjects samples (n)	Gene symbols overlapped with CIM
Myocardial Infarction	GSE48060	Peripheral blood	GPL570	21	31	*None*
	GSE83500	Aortic wall	GPL13667	20	17	*IL-6*
	GSE97320	Peripheral blood	GPL570	3	3	*HLA-B* *PPBP* *PTPRC* *SERPINA1*
	GSE61145	Serum	GPL6106	10	14	*RERE* *PADI4*
Major depressive disorders	GSE54562	anterior cingulate cortex	GPL6947	10	10	*None*
	GSE54563	anterior cingulate cortex	GPL6947	25	25	*FCGR3B* *LPA*
	GSE54564	Amygdala	GPL6947	21	21	*STAR* *ESR1*
	GSE54565	anterior cingulate cortex	GPL570	16	16	*GNB3*
	GSE54566	amygdala	GPL570	14	14	*None*
	GSE54567	dorsolateral prefrontal cortex	GPL570	14	14	*PAG1* *NSF*
	GSE54568	dorsolateral prefrontal cortex	GPL570	15	15	*ESD* *LCAT* *DMD*
	GSE54570	dorsolateral prefrontal cortex	GPL96	13	13	*None*
	GSE54571	anterior cingulate cortex	GPL570	13	13	*AR* *CNR1* *CPAMD8* *HLA-B*
	GSE54572	anterior cingulate cortex	GPL570	12	12	*MTHFR* *NCAM1*
	GSE54575	orbital ventral prefrontal cortex	GPL96	12	12	*CD4*

*MI* Myocardial Infarction, *MDD* Major Depressive Disorder, *CIM* Candidate Intermediate Molecules

**Table 2:** Differentially expressed gene or gene product suggested by the closed BITOLA system

Gene or gene product	FreqXY	FreqYZ	FreqXY*FreqYZ
LPA	1	1	1
FCGR3B	2	7	14
STAR	4	1	4
ESR1	3	2	6
GNB3	4	1	4
PAG1	1	1	1
NSF	1	1	1
ESD	1	1	1
LCAT	1	1	1
DMD	3	1	3
AR	2	1	2
CNR1	1	2	2
CPAMD8	2	4	8
HLA-B	1	1	1
MTHFR	40	4	160
CD4	11	16	176
IL6	99	20	1980
RERE	1	1	1
PADI4	1	1	1
SERPINA1	1	1	1
PTPRC	8	1	8
PPBP	4	1	4
NCAM1	1	7	7

*Freq* Frequency of co-occurrence of two concepts in literature, *X* starting concept “Myocardial infarction” Z: end concept “Major Depressive Disorder” or “Depressive disorder”

To remove the genes that were not the original ideas for the “gene or gene product”, we used the most precise method, manual checking, to evaluate the abbreviations or the alternative names for these genes used in the literatures. Fourteen genes (*FCGR3B, STAR, ESR1, PAG1, NSF, ESD, DMD, AR, CPAMD8, HLA-B, RERE, PADI4, PTPRC,* and *LPA*) failed to pass the follow-up manual literature mining inspection due to ambiguous terms aroused by the defects in the literature mining itself and thus were removed from further analysis.

### Common gene expression patterns in heart and brain tissues

**Fig. 2:**
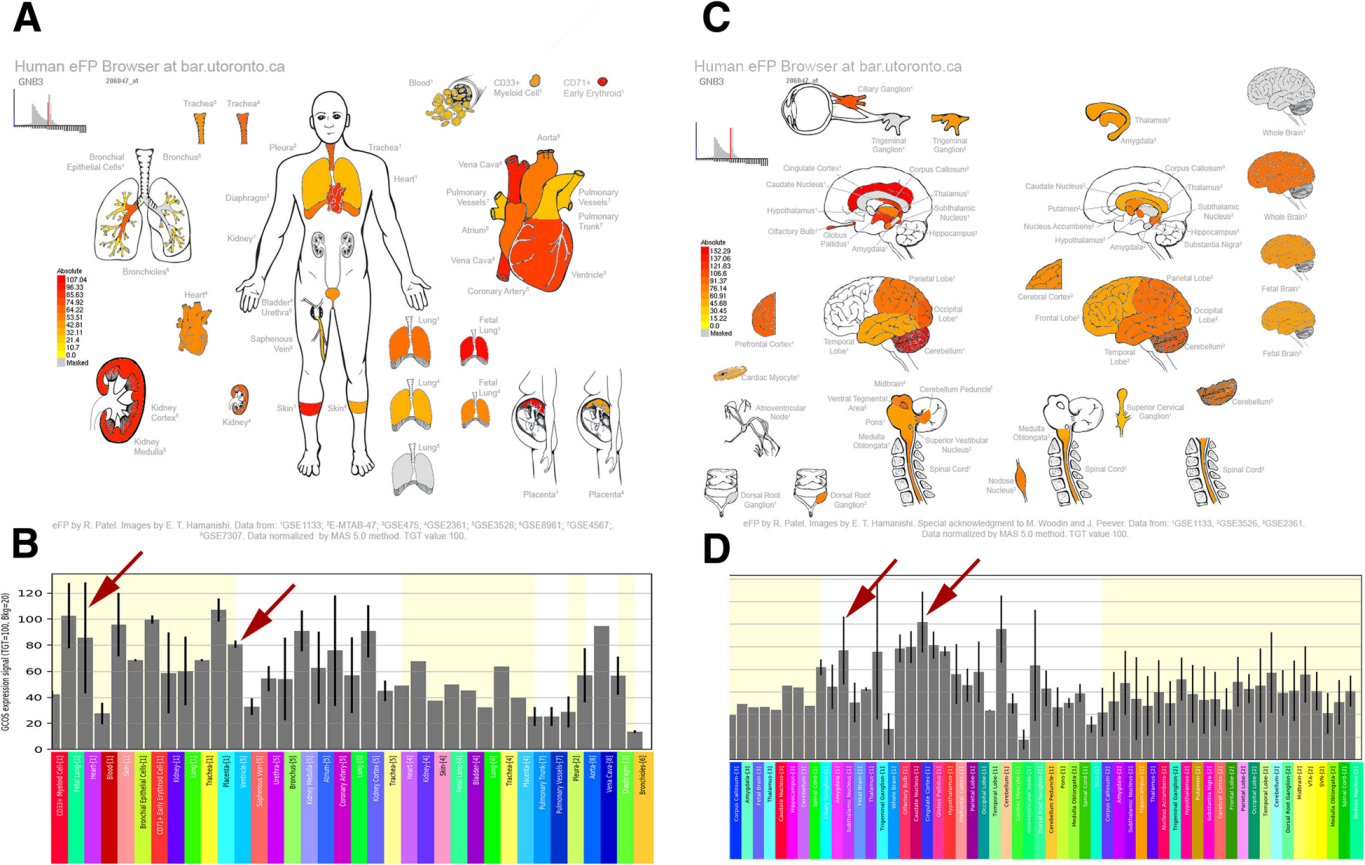
Human eFP Browser output showing *GNB3* expression in the brain and heart tissues. Strong expression levels in the heart ventricle and the cingulate cortex and subthalamic nucleus are denoted by the red colouring. **a**, **b**: Expression “anatograms” and histogram for heart tissues. **c**, **d**, Expression “anatograms” and histogram for brain tissues

**Fig. 3:**
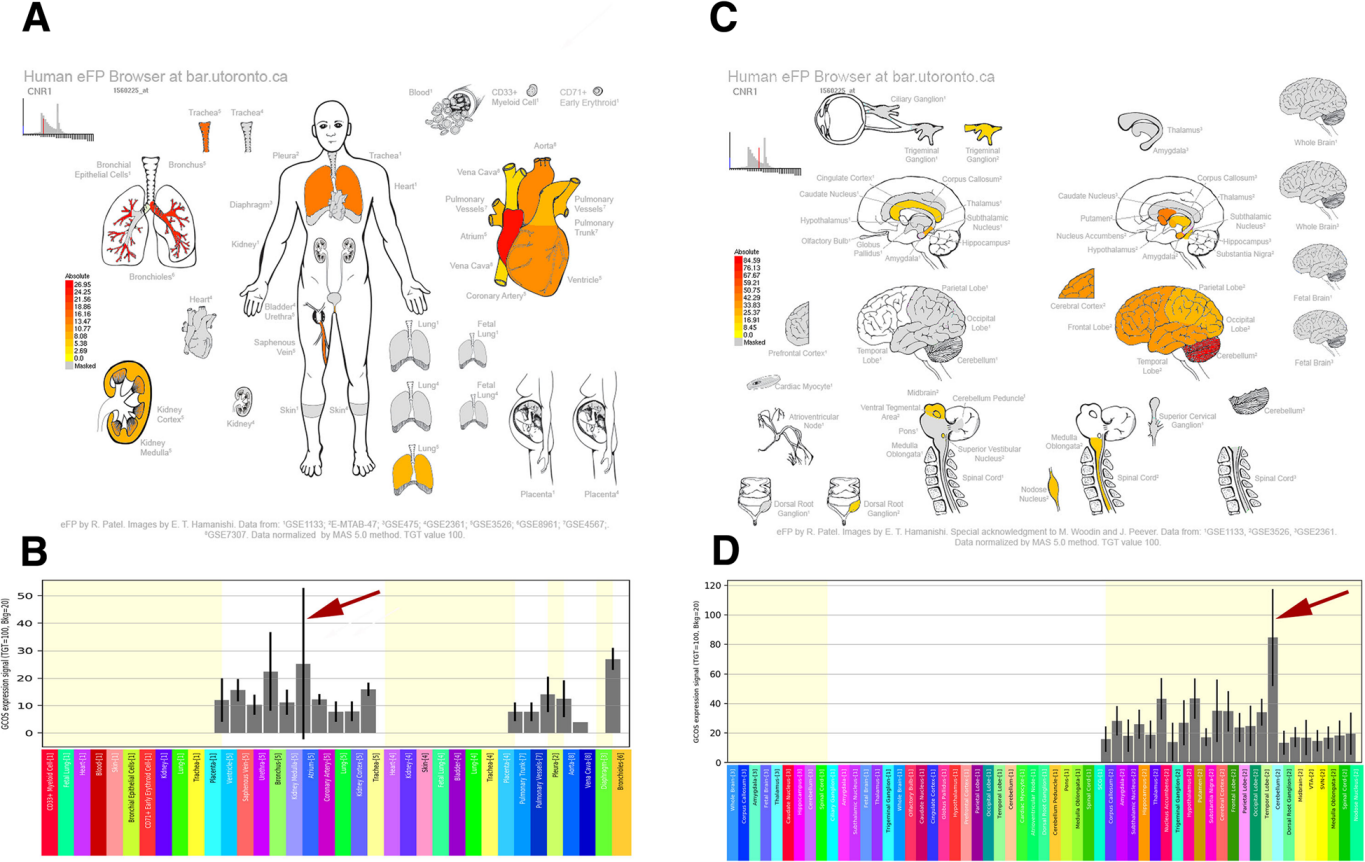
Human eFP Browser output showing *CRN1* expression in the brain and heart tissues. The highest expression areas located in the heart atrium and the cerebellum and nucleus accumbens in the brain are denoted by red colouring. **a**, **b**: Expression “anatograms” and histogram for heart tissues. **c**, **d**, Expression “anatograms” and histogram for brain tissues

**Fig. 4:**
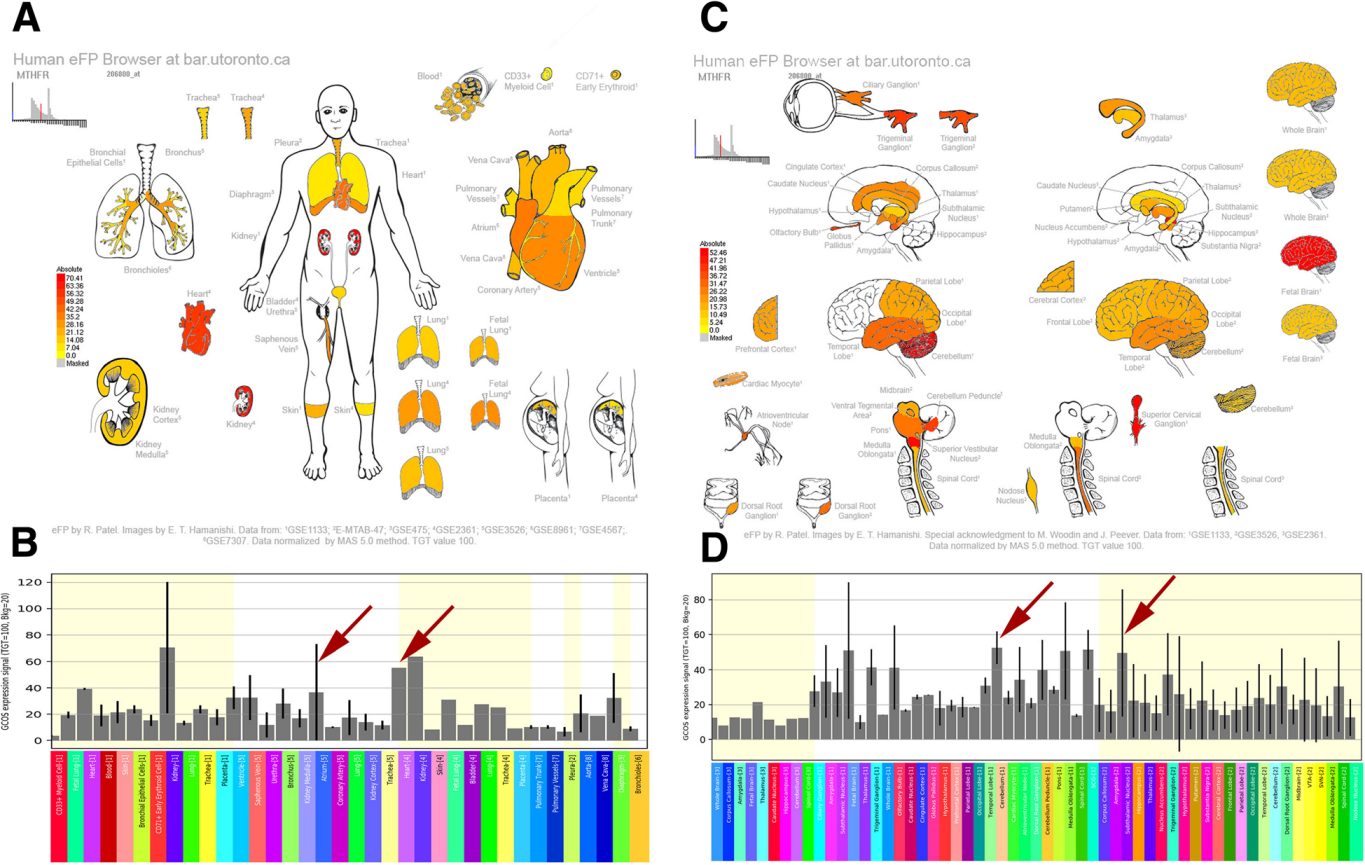
Human eFP Browser output showing *MTHFR* expression in the brain and heart tissues. High expression levels in the atrium, cerebellum, and subthalamus nucleus are denoted by red colouring. **a**, **b**: Expression “anatograms” and histogram for heart tissues. **c**, **d**, Expression “anatograms” and histogram for brain tissues

**Fig. 5:**
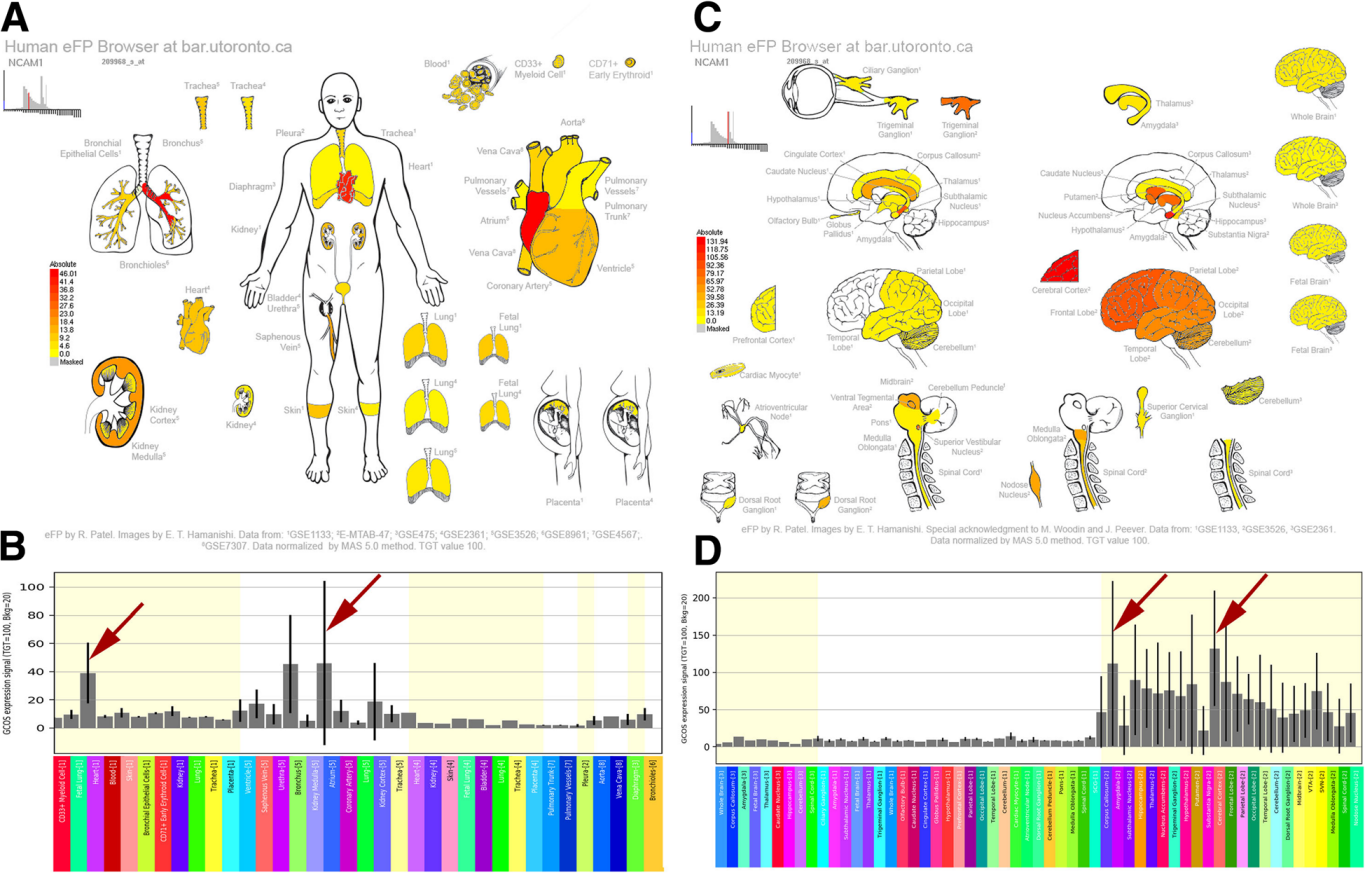
Human eFP Browser output showing *NCAM1* expression in the brain and heart tissues. Strong expression levels in the atrium, cerebral cortex, and amygdala are denoted by red colouring. **a**, **b**: Expression “anatograms” and histogram for heart tissues. **c**, **d**, Expression “anatograms” and histogram for brain tissues

In the analysis, we examined the gene expression patterns of the remaining genes by using the Human eFP Browser [23], which provides an overview of gene expression levels in the heart and brain. *LCAT, CD4, SERPINA1, IL6,* and *PPBP* failed to pass the follow-up filter, partly because these genes were not preferentially expressed in the heart tissue, which is the target of MI. Based on the tissue-specific expression patterns of the remaining genes, *GNB3, CNR1, MTHFR,* and *NCAM1* were chosen as potential candidate genes for further analysis (Fig. 2, 3, 4, 5). The analysis showed that *GNB3* was highly expressed in the heart ventricle and cingulate cortex of the brain (Fig. 2). *CRN*1 showed the highest expression in the heart atrium and cerebellum and nucleus accumbens of the brain (Fig. 3). Furthermore, *MTHFR* was overexpressed in the heart atrium and cerebellum and subthalamus nucleus of the brain (Fig. 4). Figure 5 shows the *NCAM1* gene, which has high expression in the heart atrium and cerebral cortex and amygdala of the brain. Taken together, these results suggest that the overexpression of the *GNB3, CNR1, MTHFR,* and *NCAM1* genes may contribute to the development of MI and depression and may play a role in the interaction between these two diseases.

## Discussion

In this study, we present for the first time a preliminary literature mining work exploring candidate genes related to MI and depression. By integrating data from the literature, we revealed 4 genes of interest (*GNB3, CNR1, MTHFR,* and *NCAM1*) that were likely to be associated with the aetiology of both MI and depression.

G proteins play an important role in intracellular signal transduction from the cell surface [24]. A C3T polymorphism at nucleotide 825 in exon 10 of the G protein β3 subunit gene (*GNB3/C825T*) was demonstrated to be associated with enhanced intracellular signal transduction [25] and a variety of cardiovascular risk factors, including hypertension [25], obesity [26], dyslipidaemia [27], diabetes, and atherosclerosis [28]. An association between *GNB3/C825T* and MI has also been reported [29]. In addition to the roles mentioned above, studies have implicated a role for *GNB3/C825T* in depressive disorder [30–32] and the efficacy of antidepressants for the treatment of major depression disorders [33]. In the present study, we found the highest *GNB3* expression in the heart ventricle and cingulate cortex of the brain (Fig. 2), which was in accordance with the aetiology of depression [34] . Thus, further study of *GNB3* is essential for assessment of the interaction between MI and depression.

Cannabinoid receptor 1 (*CNR1*) is one member of the seven transmembrane G-protein coupled receptor family and can regulate the levels of second messenger mainly through coupling with G proteins after activation by endocannabinoids [35, 36]. The *CNR1* receptor may play a protective role through a wide variety of mechanisms, including inhibition of excessive noradrenaline release from the sympathetic nerve fibres [37], lowering inflammation, oxidative stress, fibrosis, and excitotoxicity, and enhancing blood flow [38]. Therefore, cannabinoid receptor agonists can be considered as a prospective group of compounds for creation of drugs that are able to protect the heart against ischaemia-reperfusion injury in the clinical setting [39]. Over the past few years, numerous studies have suggested that depression directly results in the hyperactivity of the hypothalamic-pituitary-adrenal axis [6]. Studies have also suggested that *CNR1* negatively regulates the hypothalamic-pituitary-adrenal axis function [40, 41]. In addition, mice lacking *CNR1* can develop depressive-like behaviours or disorders [42]. Specifically, in our study, high *CNR1* expression in the brain areas was observed at the nucleus accumbens (Fig. 3), which has been suggested to be related to a lack of interest and other symptoms of depression [43]. The evidence above suggests that targeting the endocannabinoid system may evolve as a novel therapeutic concept to limit the devastating consequences of MI and depression.

Methylenetetrahydrofolate reductase (*MTHFR*) is a key enzyme involved in homocysteine metabolism. An elevated total plasma homocysteine level has been demonstrated to be associated with both cardiovascular disease and depression [44, 45]. Because the C-to-T transition can cause reduced enzyme activity and elevated total plasma homocysteine levels, a positive relationship may exist between the *MTHFR* 677 C → T polymorphism and these two diseases, which has also been demonstrated [46, 47]. This polymorphism was also associated with a risk of MI [48, 49]. Moreover, the results confirmed those of very recent meta-analyses of genome-wide association studies, suggesting that *MTHFR* was a genetic overlap candidate gene that likely was shared between mood disorders and cardiovascular diseases [50]. These findings provide some concrete directions for further research.

*NCAM1*, which is also known as *CD56*, is a member of the immunoglobulin superfamily [51]. *NCAM1* was first identified in brain tissue and is the best surface antigen for identification of human NK cells [52]. Numerous studies have suggested that *NCAM1* is a gene of interest associated with the pathogenesis of depressive disorder [52–54]. Experimental evidence showed that *NCAM* deficiency in mice resulted in a depression-like phenotype that could be reversed by an *NCAM*-derived peptide [55]. In the present study, the *NCAM1* gene was mainly expressed in the cerebral cortex and amygdala in the brain (Fig. 5), which are involved in the pathogenesis of depression [56]. In addition to its role in depression, studies have also suggested its correlations with MI [57]. One study demonstrated that *NCAM1* was upregulated under metabolic stress in cardiomyocytes and suggested that *NCAM1* was a cardioprotective factor [58]. Hence, this evidence may have implications for the role of *NCAM1* in communication between MI and depression that warrants further exploration.

## Conclusion

In conclusion, using literature mining methods, the *GNB3, CNR1, MTHFR,* and *NCAM1* genes were identified and directly or indirectly implicated in the regulation of MI and depression. Although additional research is needed to confirm these findings, our study reduced the candidate causal genes to a manageable number and might present potential new clues for future research.

## Data Availability

The data are available at: http://arnika.mf.uni-lj.si/pls/bitola2/bitola and http://bar.utoronto.ca/efp_human/.

## Abbreviations

BITOLA: Biomedical discovery support system
CIM: Candidate intermediate molecule
*CNR1*: Cannabinoid receptor 1
DEGs: Differentially expressed genes
GEO: Gene Expression Omnibus
*GNB3*: G protein β3 subunit gene
MI: Myocardial infarction
*MTHFR*: Methylenetetrahydrofolate reductase
*NCAM1*: Neural cell adhesion molecule 1
